# Poly(3-hydroxybutyrate) production in an integrated electromicrobial setup: Investigation under stress-inducing conditions

**DOI:** 10.1371/journal.pone.0196079

**Published:** 2018-04-26

**Authors:** Israa Salem Al Rowaihi, Alexis Paillier, Shahid Rasul, Ram Karan, Stefan Wolfgang Grötzinger, Kazuhiro Takanabe, Jörg Eppinger

**Affiliations:** 1 King Abdullah University of Science and Technology, KAUST Catalysis Center, Physical Sciences and Engineering Division, Thuwal, Kingdom of Saudi Arabia; 2 Technical University of Munich, Department of Mechanical Engineering, Institute of Biochemical Engineering, Garching, Germany; 3 King Abdullah University of Science and Technology, Computational Bioscience Research Center, Biological and Environmental Sciences and Engineering Division, Thuwal, Kingdom of Saudi Arabia; Karl-Franzens-Universitat Graz, AUSTRIA

## Abstract

Poly(3-hydroxybutyrate) (PHB), a biodegradable polymer, can be produced by different microorganisms. The PHB belongs to the family of polyhydroxyalkanoate (PHA) that mostly accumulates as a granule in the cytoplasm of microorganisms to store carbon and energy. In this study, we established an integrated one-pot electromicrobial setup in which carbon dioxide is reduced to formate electrochemically, followed by sequential microbial conversion into PHB, using the two model strains, *Methylobacterium extorquens* AM1 and *Cupriavidus necator* H16. This setup allows to investigate the influence of different stress conditions, such as coexisting electrolysis, relatively high salinity, nutrient limitation, and starvation, on the production of PHB. The overall PHB production efficiency was analyzed in reasonably short reaction cycles typically as short as 8 h. As a result, the PHB formation was detected with *C*. *necator* H16 as a biocatalyst only when the electrolysis was operated in the same solution. The specificity of the source of PHB production is discussed, such as salinity, electricity, concurrent hydrogen production, and the possible involvement of reactive oxygen species (ROS).

## Introduction

Biodegradable plastics are gaining wide interest. They are derived from inexpensive biomaterial, hence represent a sustainable and environmentally safe alternative to the synthetic petroleum-based polymers [1, 2], which are introduced in substantial amounts into the ecosystem as residential and industrial waste products [3–5]. The most common biodegradable plastic is poly(3-hydroxybutyrate) (PHB) that belongs to the family of polyhydroxyalkanoate (PHA) [6]. PHB is a highly reduced carbon storage compound that serves as carbon and energy reserve in different microorganisms. In these microorganisms, it is synthesized from acetyl-CoA through the successive action of three enzymes, namely the *β*-ketoacyl-CoA thiolase (*phb* A), the acetoacetyl-CoA reductase (*phb* B) and the PHB polymerase (*phb* C) that polymerizes acyl coenzyme A (acyl-CoA). The bacteria utilize PHB when nutrients are limited through depolymerization into 3-hydroxybutyric acid (3HB) which is used to produce acyl-CoA and acetyl-CoA, that is metabolized in the tricarboxylic acid (TCA) cycle as a source of carbon and energy [4, 7]. PHB is produced in native and engineered microorganisms by accumulating as granules in the cytoplasm in response to conditions of physiological stress. Cells with high PHB content had enhanced survival and tolerance toward heat challenge and oxidative stress [8–10]. A study using recombinant *E*. *coli* reported an increase in heat resistance with PHA production compared with the control strain [11]. Another study using *Aeromonas hydrophila* suggested the enhancement of the survival ability of the strain by the simultaneous biosynthesis of PHA granules under various stress conditions [12]. A third study that exposed knock out mutants of *Burkholderia* strains lacking essential genes for PHA production, to nutrient limitations, high osmotic pressure and high temperature showed lower survival compared to the wild-type [12].

The knowledge of stress tolerance enhancements by PHB accumulation in microorganisms was further investigated by Goh *et al*.,[6]. This study showed that a modified *E*. *coli* strain (harboring the PHB synthesis genes) increases PHB production to protect cells from oxidative damage caused by reactive oxygen species (ROS) such as hydroxyl radicals (OH•), superoxide anion (O_2_^−^), and hydrogen peroxide (H_2_O_2_). They also reported the role of 3HB (the monomer of PHB) as a specific site for binding of stress-resistant proteins, which enhances bacterial stress tolerance.

**Fig 1:**
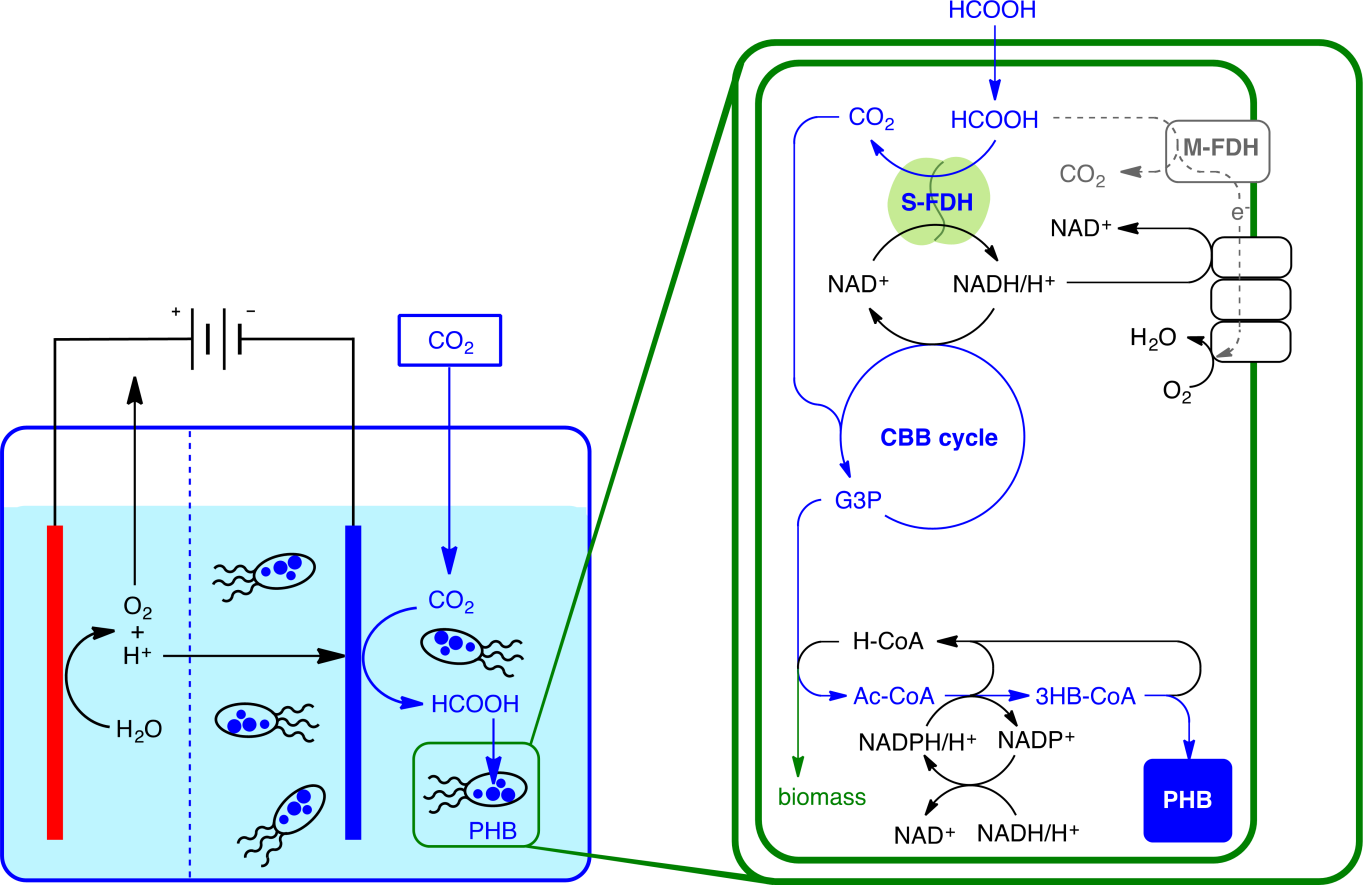
Schematic overview of the one-pot IEMC (Integrated Electro-Microbial Carbon Capture) harvesting energy from renewables for the electrolytic reduction of CO_2_ into formate, then the microbial conversion of formate into PHB through the CBB cycle. M-FDH: membrane formate dehydrogenase, S-FDH: soluble formate dehydrogenase, CBB: Calvin Benson Cycle, 3GP: 3 glyceraldehyde phosphate.

In this contribution, we investigated the influence of different stressors on PHB synthesis in an 8-h investigation cycle. We used the two well studied PHB production strains; *Methylobacterium extorquens* AM1 and *Cupriavidus necator* H16 (previously known as *Ralstonia eutropha* H16 [13]). These strains were exposed to stress conditions caused by a combination of electrolysis, relatively high salinity, nutrient deficiency and conditions including nitrogen depletion or limitation. Formate was selected as the carbon source for the microorganisms based on the literature showing pure PHB production [14]. We selected a suitable setup to analyze these stressors by using an Integrated Electro-Microbial Carbon Capture setup (IEMC) (Fig 1), where formate and electrolysis were generated from the electrochemical reduction of carbon dioxide (CO_2_) in a comparatively high salinity buffer [15, 16]. Furthermore, we monitored the growth, PHB production and formate uptake.

## Materials and methods

### Materials and chemicals

All materials, salts and other chemicals mentioned below were purchased from Sigma-Aldrich and used as received.

### Microorganisms

Microbial strains (*Cuprividus necator* H16, or *C*. *necator*, DSM 428 and *Methylobacterium extorquens* AM1, or *M*. *extorquens*, DSM 1338) were purchased from the German Collection of Microorganisms and Cell Cultures (DSMZ, Braunschweig, Germany). Fresh cultures were prepared from aliquots stored at −80°C.

### Pre-cultures

Pre-cultures of *C*. *necator* were prepared from frozen stocks that were grown overnight in rich medium (1 L) consisting of peptone (5 g L^−1^) and meat extract (3 g L^−1^) and were then sub-cultured (10%, v/v) in minimal medium ‘R’ (1 L) consisting of (NH_4_)_2_SO_4_ (3 g L^−1^), KH_2_PO_4_ (1.5 g L^−1^), Na_2_HPO_4_ (4.45 g L^−1^), MgSO_4_ (0.097 g L^−1^), CaCl_2_•6H_2_O (0.02 g L^−1^), FeSO_4_•7H_2_O (0.02 g L^−1^), MnCl_2_•4H_2_O (24 μg L^−1^), ZnSO_4_•7H_2_O (528 μg L^−1^), Na_2_MoO_4_•2H_2_O (150 μg L^−1^), CuSO_4_•5H_2_O (240 μg L^−1^), CoCl_2_•6H_2_O (90 μg L^−1^), H_3_BO_3_ (864 μg L^−1^), NiCl_2_ (24 μg L^−1^), and formic acid (30 mM) as a carbon source. The sub-cultures were incubated in an incubator shaker at 30°C and 140 rpm (Innova 42, Eppendorf, Hamburg, Germany). Microbial growth was determined by measuring the optical density at 600 nm (OD_600_), while using an uninoculated media as a blank (Novaspec III Amersham Biosciences, Buckinghamshire, United Kingdom).

Pre-cultures of *M*. *extorquens* were prepared from frozen stocks that were grown overnight in rich medium (1 L) consisting of peptone (5 g L^−1^) and meat extract (3 g L^−1^) with the addition of 1% (v/v) methanol, and were then sub-cultured (10%, v/v) in minimal medium ‘M’ (1 L) consisting of, (NH)_4_SO_4_ (1.5 g L^−1^), NH_4_Cl (1.5 g L^−1^), NH_4_NO_3_ (0.15 g L^−1^), KH_2_PO_4_ (1.3 g L^−1^), K_2_HPO_4_ (0.176 g L^−1^), NaH_2_PO_4_ (0.77 g L^−1^), Na_2_HPO_4_ (1.2 g L^−1^), MgSO_4_ (0.05 g L^−1^), CaCl_2_•6H_2_O (0.03 g L^−1^), FeSO_4_•7H_2_O (0.02 g L^−1^), MnSO_4_•7H_2_O (500 μg L^−1^), ZnSO_4_•7H_2_O (3 mg L^−1^), Na_2_MoO_4_•2H_2_O (80 μg L^−1^), CuSO_4_•5H_2_O (80 μg L^−1^), CoCl_2_•6H_2_O (800 μg L^−1^), H_3_BO_3_ (600 μg L^−1^) and formic acid (30 mM) as a carbon source.

### Preparation and characterization of indium nanoparticle electrodes (In-NP)

An indium metal plate (0.25 mm and 99.999% metal basis) was washed briefly with 0.1 M HCl, followed by electrochemical deposition of indium from an aqueous 0.05 M In_2_(SO_4_)_3_ solution in 0.04 M citric acid. Using another indium metal substrate as a counter electrode, In-NP electrode was fabricated on an In plate by applying a galvanometric current of −9.3 mA cm^−2^ for 10 min. Field emission scanning electron microscopy (FE-SEM) (Nova NanoSEM, FEI, Hillsboro, Oregon, USA) was used to investigate the size and morphology of the electrodes.

### Electrochemical CO_2_ reduction

A custom made electrochemical cell (S1 Fig) equipped with a potentiostat (BioLogic VMP3, Paris, France) was employed. Three electrodes were used to monitor the current-potential response of the working electrode. A Pt wire and an Ag/AgCl electrode (in saturated KCl) were employed as the counter electrode and reference electrode, respectively. In-NP electrode was used as the working electrode (1.7 cm^−2^). The applied potential was converted to a reversible hydrogen electrode (RHE). Before the electrolysis, the electrolyte (50 mL) was saturated with CO_2_ for 1 h. The chamber was placed in a water bath to maintain the chamber temperature at 30°C. For agitation, a small magnetic stirrer (5 mm) was placed in the chamber at 140 rpm. A continuous flow of CO_2_ was maintained at a flow rate of 10 mL min^−1^. The experiments were performed in duplicates.

### Identification of products formed during the electrochemical CO_2_ reduction

To confirm the identities and quantities of the liquid and gas phase products during electrochemical reactions, high-performance liquid chromatography (HPLC, Agilent 1200 series, California, USA) and online micro gas chromatography (T-3000, SRI instruments, Lyon, France) were employed, respectively. To quantify the liquid products, the HPLC was equipped with an ICE-Coregel 87 H3 column (Transgenomic, Apple Valley, Minnesota, USA), and the eluent was 0.008 N H_2_SO_4_ solution at a flow rate of 0.8 mL min^−1^, using a UV-VIS detector at 214 nm and 35°C. For analysis of the gaseous products, a MolSieve 5A column attached to a thermal conductivity detector (TCD) was used (T-3000, SRI instruments, Lyon, France). The minimum detection limit for gaseous products was 50 ppm. The measurements were performed in duplicates.

### Integrated Electro-Microbial carbon Capture experiments (IEMC)

**Fig 2:**
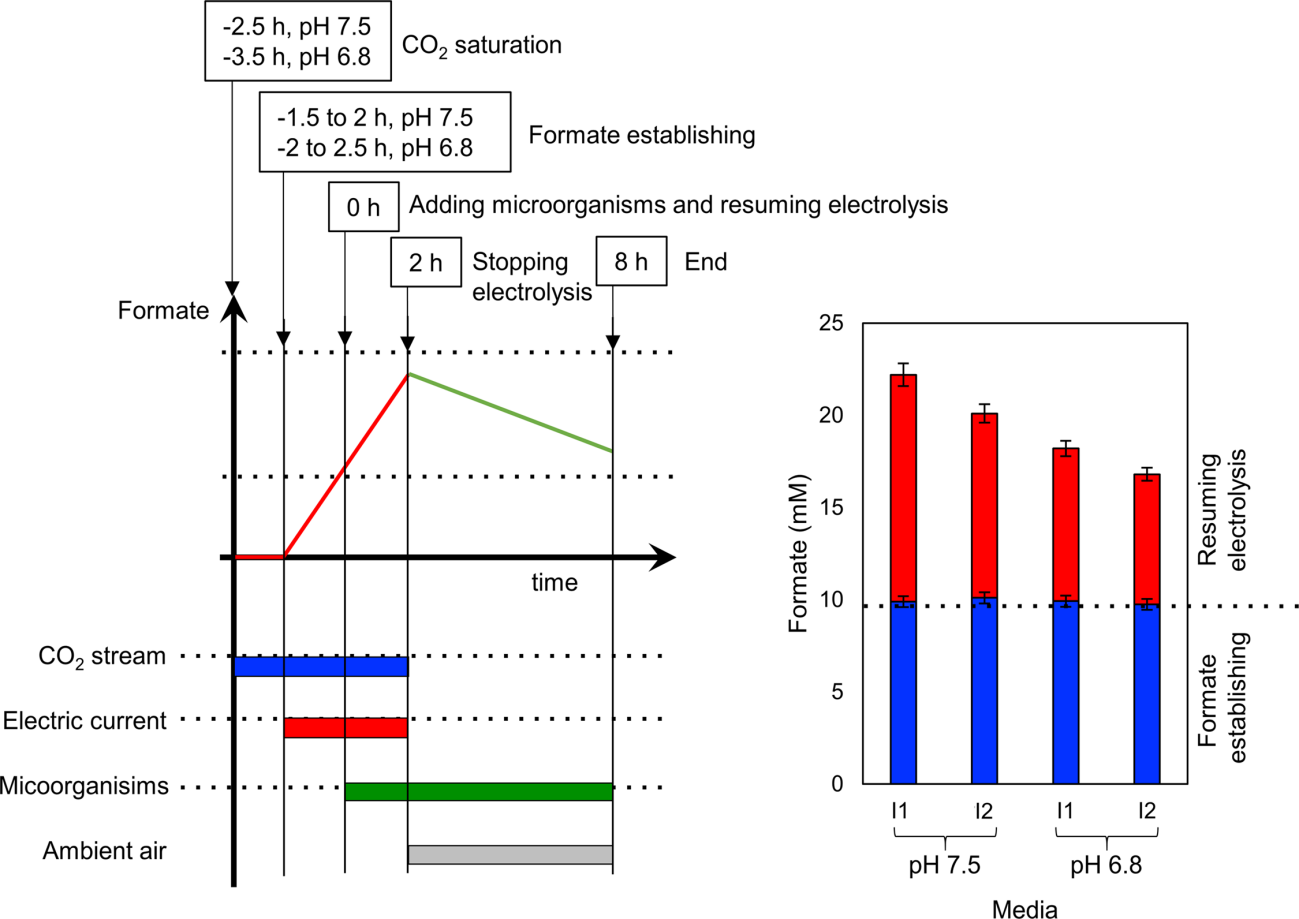
Schematic overview of the experimental procedure, along with the electrolysis profile, and formate concentration during the IEMC experiments (50 mL, media I1 or I2 at pH 7.5 for cultures of *C*. *necator*, or at pH 6.8 for cultures of *M*. *extorquens*). **Left figure:** Electrolysis profile prior to adding the microorganisms with a potential of −1.2 V vs. RHE (red bar, formate establishing), in CO_2_ saturated medium with a constant CO_2_ gas flow of 10 mL min^−1^ (blue bar, CO_2_ saturation) under stirring (140 rpm). At 0 h the microorganisms were added (green bar, adding microorganisms and resuming electrolysis) and the electrolysis was continued for another 2 h, where the formate concentration increased (red line, 2 h). After electrolysis (2 h), the microorganisms were grown under ambient air conditions (grey bar, stopping electrolysis) for 6 h (total of 8 h run). The growth, formate uptake (green line, 8 h) and PHB production were measured. **Right figure:** Establishment of formate concentrations in media I1 or I2 (blue bar up to 0 h) and the measured formate concentration after resuming electrolysis (red bar, from 0 to 2 h).

The setup and sequence are illustrated in Figs 1 and 2. Medium ‘I1’ consists of 40 mM K_2_HPO_4_ and 100 mM K_2_SO_4_ solution, resulting in nitrogen and trace metals (TM) deprivation conditions. Medium ‘I2’ consists of 40 mM K_2_HPO_4_, 100 mM K_2_SO_4_ solution and 3% (v/v) of medium C (cultures of *C*. *necator*) or M (cultures of *M*. *extorquens*), TM and nitrogen limited conditions. Media I1 or I2 (50 mL) was adjusted to pH 7.5 (cultures of *C*. *necator*) or pH 6.8 (cultures of *M*. *extorquens*). A volume of 50 mL of the resulting solution was transferred into the electrolysis cell described above (Fig 1) and saturated with a continuous flow of CO_2_ gas (99.9999%, 10 mL min^−1^) under stirring (140 rpm). After 1 h for CO_2_ saturation, a potential of −1.2 V vs. RHE was applied while the CO_2_ flow and stirring speed were maintained and the current stabilized at 10 mA cm^−2^ (Fig 2, left). Throughout the experiment, the pH was monitored hourly and maintained within 0.2 pH units by addition of small quantities of 4 M HCl or 2 M KOH. After approximately 2 h, when the formate concentration reached 10 mM, cells were transferred from pre-cultures into the electrolysis cells as described above. After 2 h of the inoculation, the CO_2_ stream and electric current were discontinued; the electrochemical cell was flushed with air and kept open towards the atmosphere while stirring continued. Formate concentration and OD_600_ were monitored every 2 h. After 6 h (total of 8 h culturing), cells were harvested by centrifugation and the PHB concentration was determined from the collected and washed cell pallet as described below. The experiments were performed in a set of duplicates.

### PHB extraction

Culture samples (30 mL) were centrifuged (Eppendorf centrifuge 5430 R, Eppendorf, Hamburg, Germany) at 6,500 × g for 50 min at 4°C and the cell pellets were washed with double-distilled H_2_O to decrease the residual salts from the medium. Subsequently, cell pellets were re-suspended in water, flash frozen in liquid N_2_, and lyophilized. The lyophilized cell pellets (approximately 30 mg) were dissolved in 2 mL of 6% (v/v) sulfuric acid in a methanol solution containing 100 mg L^−1^ of sodium benzoate as an internal standard. Then, 2 mL of chloroform was added, and the mixture was heated for 3 h at 100°C in a tightly sealed pressure tube placed in an oil bath (Heating bath for oil and hot Plate Stirrer HE-036110550, Heidolph, Chicago, USA). After methanolysis, the samples were cooled on ice for 10 min, and 1 mL of double-distilled H_2_O per 2 mL of CHCl_3_ (1:2 ratio) was added [17]. The mixture was vortexed for 1 min, and the phases were separated by centrifugation (Eppendorf centrifuge 5430 R, Eppendorf, Hamburg, Germany) at 4,500 × g for 5 min at 4°C. The organic phase was collected, neutralized with 0.25 g (to 2 mL) NaHCO_3_ and dried over with 0.25 g Na_2_SO_4_ [18].

### GC analysis and quantification of PHB production

The resulting mixtures of 3-hydroxy-butanoyl methylester (3HBM) and intracellular components in CHCl_3_ were characterized and quantified by GC/FID (Agilent Technology GC system 7890A/5975 inter XL EI, CI MSD with triple-axis detector, California, USA). A standard curve was determined from a commercial poly[(R)-3-hydroxybutyric acid] standard (Sigma-Aldrich). The GC/FID was equipped with a DB-WAX column (60 m × 0.25 μm × 0.5 μm, Agilent Technologies, California, USA) and sample components were separated using a temperature profile of 50°C for 1 min, and temperature was increased to 240°C at a ramping rate of 15°C min^−1^, and hold for 5 min (run time: 18.6 min). The injection volume was 1 μL. The flow rate was 1.7 mL min^−1^ [18]. The measurements were performed in duplicates.

## Results

The PHB production was attempted using IEMC setup, being placed the microorganisms under comparatively high salinity, which essentially reduces solution resistance for electrolysis for initial formate production. To analyze the effects of different stressors on cell growth and PHB production, the IEMC experiments were conducted at limited carbon source (formate), relatively high salinity, along with nitrogen and trace metals (TM) limitation (I2 medium) or nitrogen and TM deprivation (I1 medium).

As the first step of the IEMC approach, the formation of formate from CO_2_ using In electrode was separately investigated in the I1 medium. The In nanoparticle decorated In electrode (In-NP) improved the current density and Faradaic efficiency compared to the bare In electrode (S1 Text, S2 and S3 Figs). The improvement is reasonably ascribable to enhancement in the active surface area and minimizing unselective sites that were prevalent for the bare In plate electrode. The In-NP electrode reached a current density of −10 mA cm^−2^ at an applied potential of −1.2 V vs. RHE with a formate production rate of 3.2 mM h^−1^ cm^−2^ (see also S4 Fig for the results at different applied potentials). This corresponds to a Faradaic efficiency of 86% for formate with an energy efficiency for the CO_2_ reduction to formate of 37.5%.

**Fig 3:**
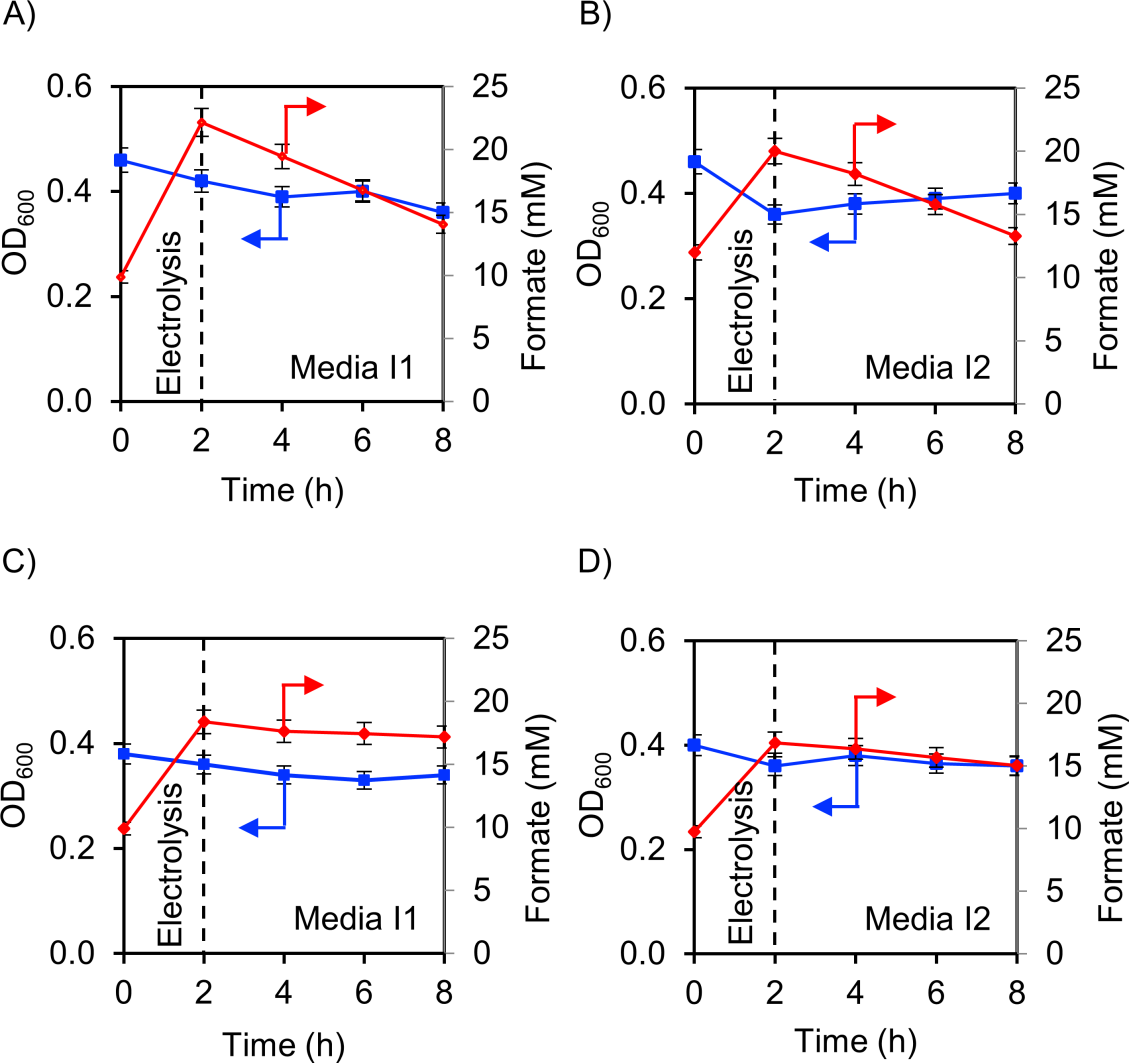
IEMC results with media I1 and I2. Electrochemical reaction (working electrode: In, reference electrode: Ag/AgCl, CE: Pt wire (in an isolated room), −1.2 V vs. RHE, CO_2_ saturated, 293 K, 40 mM phosphate, 100 mM K_2_SO_4_). Pre-run before integration (−2 to 0 h) produced 10 mM formate. After 2 h the cell was vented with ambient air. A) formate (red) and growth profile (blue) for *C*. *necator* at pH 7.5 in media I1. B) formate and growth profile for *C*. *necator* at pH 7.5 in media I2. C) formate and growth profile for *M*. *extorquens* at pH 6.8 in media I1. D) formate and growth profile for *M*. *extorquens* at pH 6.8 in media I2. FA: formate and OD_600_: growth measurement.

As the second step, the microbial formate consumption, growth and PHB production were monitored for both model strains in IEMC conditions in media I1 (nitrogen and TM deprivation with relatively high salinity) and I2 (nitrogen and TM limitation with relatively high salinity). In the setup, electrolysis was conducted (1.5 to 2 h at pH 7.5, and 2 to 2.5 h at pH 6.8) to establish a concentration of 10 mM formate in the reactor, prior to the addition of the microbial cells. Following the cell addition, a second electrolysis was performed for 2 h, subjecting the microorganisms to electrolysis, and to increase the formate concentration (Fig 2). During this period, the electrolysis resulted in an increase in formate concentration (Fig 2, right) from 10 mM (at 0 h) to 22.2 mM in medium I1 and 20.1 mM in medium I2 at pH 7.5 for *C*. *necator*. For the *M*. *extorquens* media, where a more acidic pH of 6.8 was required, the amount of formate produced was 18.2 mM in medium I1 and 16.8 mM in medium I2. Therefore, at a lower pH, less formate was produced. The production of formate was generally lower in medium I2 compared to I1 (Fig 2, right). Fig 3 compiles the results for the formate concentrations and the microbial growth (OD_600_) during the IEMC experiments. During electrolysis, the OD_600_ noticeably decreased by 9% in medium I1 and 21% in medium I2 for *C*. *necator* and 5% in medium I1 and 10% in medium I2 for *M*. *extorquens*. For both microorganisms, the reduction in OD_600_ was more pronounced in media I1.

After the electrolysis, the IEMC cell was vented with ambient air (Fig 2 at the 2 h mark, and in Fig 3 indicated by dotted lines). The introduced oxygen should allow ATP synthesis, required for PHB production through oxidative phosphorylation (Eq 1) [15, 19].

$$1.3\;\text{NADH/H}+\frac{1}{2}{\text{O}}_{2}+2.7\;\text{ADP}+2.7{\;\text{P}}_{\text{i}}\to {\text{NAD}}^{+}+2.7\;\text{ATP}+{\text{H}}_{2}\text{O}$$ Eq 1

**Fig 4:**
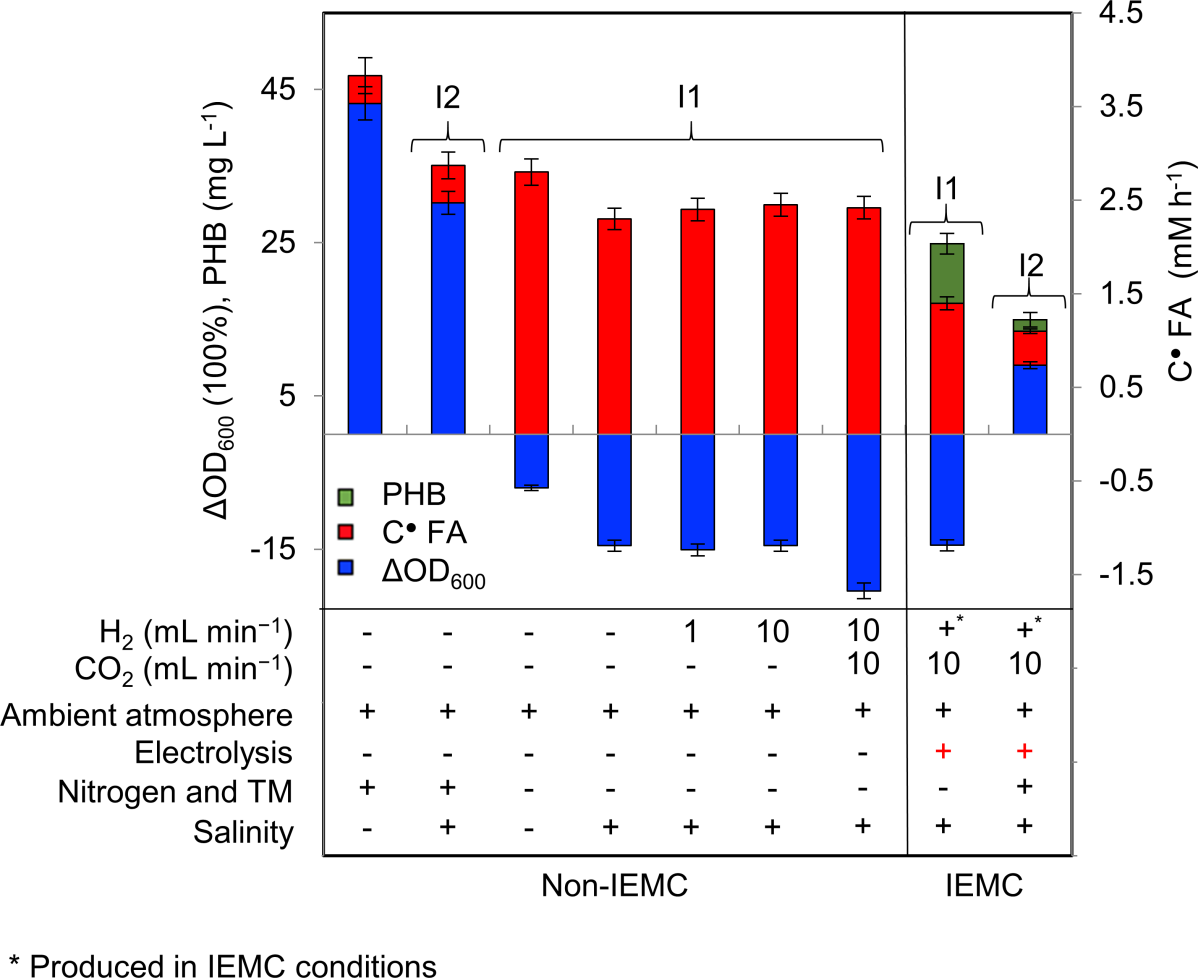
Screening profile at pH 7.5 of *C*. *nector* for growth, formate and PHB production in IEMC and non-IEMC conditions in I1 and I2 media. Under non-IEMC conditions, we used control experiments to test the influence of trace metals (TM) (3% (v/v) of medium C), salinity (100 mM K_2_SO_4_) and varying gas flows of H_2_ and CO_2_, on the investigated parameter and compared them to IEMC conditions where electrolysis is applied. The flow of H_2_ and CO_2_ had no influence on growth or PHB production. The addition of TM resulted in growth. The salinity caused growth inhibition but adding TM with salinity counters this effect strongly. Adding TM in IEMC promoted growth, but not as high as non-IEMC. PHB was only detected under electrolysis conditions. The formate uptake was reduced in IEMC compared to non-IEMC. ċ_FA_: formate uptake rate, Δ OD_600_: growth measurements.

The formate uptake rate (after 8 h) for *M*. *extorquens* was 0.3 mM h^−1^ in medium I1 and 0.2 mM h^−1^ in medium I2 (Fig 3) at an OD_600_ of 0.4, which was very low compared to *C*. *necator* with approximately 1.3 mM h^−1^ and 1.1 mM h^−1^, respectively, at an OD_600_ of 0.45. The formate uptake for *C*. *necator* was reduced under IEMC conditions compared to non-IEMC conditions (Fig 4, red bars). There was no recovery in growth in all experiments (Fig 3A, 3C, and 3D), except the slight recovery for *C*. *necator* in medium I2 (Fig 3B). This result is intriguing because, in medium I2, *C*. *necator* showed substantial growth under non-IEMC conditions (Fig 4, medium I2). The absence of growth in medium I1 under IEMC conditions and non-IEMC conditions was independent of bubbling with hydrogen or CO_2_, due to the absence of nitrogen and TM (Figs 3A and 4, media I1).

*M*. *extorquens* did not show PHB production, neither by adding formate nor running the IEMC (in media I1 and I2). Also, *C*. *necator* did not show PHB production when formate was added to the media (in media I1 and I2, Fig 4, non-IEMC). Interestingly, only when electricity was applied, *C*. *necator* showed PHB production (Fig 4, IEMC). The PHB production of *C*. *necator* during the IEMC run resulted in 25.2 mg L^−1^ in medium I1 (1.3 mM h^−1^ formate uptake) and 13.0 mg L^−1^ in medium I2 (1.1 mM h^−1^ formate uptake) (Fig 4, IEMC).

To investigate the potential role of electrochemically produced hydrogen during electrolysis in the formation of PHB, control experiments were performed, without electrolysis. Therefore, the microorganisms were cultured in medium I1in the identical electrochemical cell and 30 mM formate were added as a carbon source. During the first two hours of culturing, H_2_ and CO_2_ were fed to the setup with the following flow rates: 1 to 0, 10 to 0, and 10 to 10 mL min^−1^ of H_2_ to CO_2_, respectively. In these IEMC analog experiments, the rate of formate uptake and growth reduction were similar to those observed for aerobic cultures (Fig 4, non-IEMC), but no PHB production was detected.

## Discussion

A basic amount of salinity was required for electrochemical conversion of CO_2_. However, this salt addition generally led to a decrease of cell population and formate uptake compared to non-salinity control conditions for *C*. *necator* (Fig 4, left most column). This effect could partially be rescued by adding nitrogen and trace metals (Fig 4, media I2), and their absence can severely affect growth (Fig 4, media I1). This reduced decline in cell amount can be explained by providing essential components for production and function of metalloproteins [20]. On the contrary, the addition of both components led to depositions on the cathode and an increased formation of H_2_ as shown during IEMC conditions (S5 Fig). But the presence of relatively high salinity does not seem to influence PHB production (Fig 4) for *C*. *necator*.

**Fig 5:**
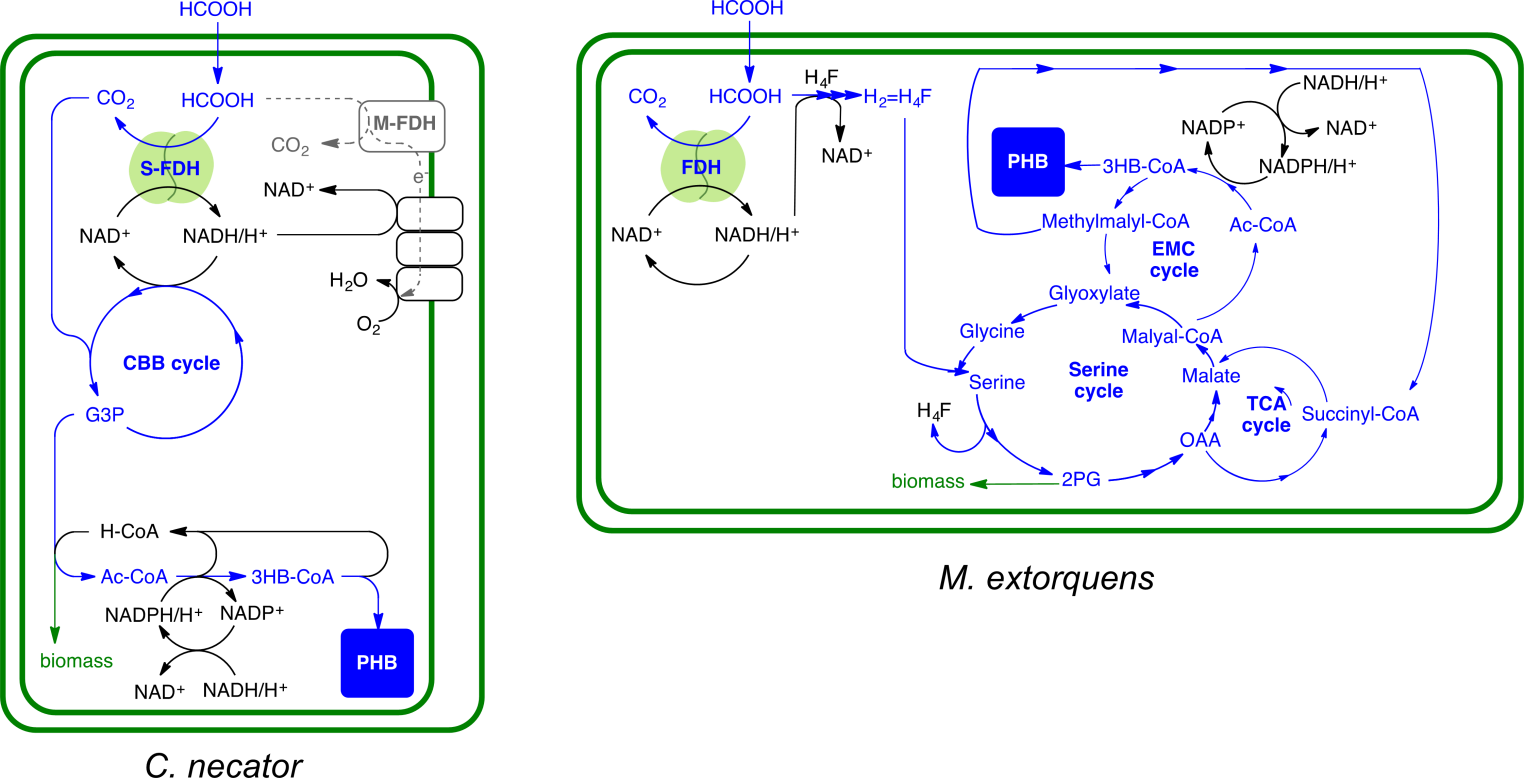
Metabolic pathways of *C*. *necator* compared to *M*. *extorquens* for PHB production from formate. *C*. *necator* (left) reduces formate into CO_2_, then captures it through the CBB cycle to produce PHB (M-FDH: membrane formate dehydrogenase, S-FDH: soluble formate dehydrogenase, CBB: Calvin Benson Cycle, 3GP: 3 glyceraldehyde phosphate). *M*. *extorquens* (right) however, condenses formate with tetrahydropterin (H_4_F) and gives formyl-tetrahydrofolate that is further converted to methylenetetrahydrofolate (H_2_ = H_4_F). The later condenses with glycine in the serine cycle. The glycine is produced from glyoxylate that is regenerated from in the ethylmalonyl-CoA cycle (EMC) from acetyl-CoA (Ac-CoA) assimilation. From the EMC cycles, PHB branches out, using 3HB-CoA (3 hydroxybutyrate-CoA). The EMC cycle also directs the Ac-CoA for methylmalys-CoA production that is required by the TCA cycle.

*M*. *extorquens* did not produce PHB during IEMC conditions, whereas *C*. *necator* produced significant levels of PHB (Fig 4, IEMC conditions). This difference is most likely based on the fact that *M*. *extorquens* uses the different and more complex serine cycle for the conversion of formate to PHB [21], whereas *C*. *necator* uses the CBB cycle (Calvin-Benson-Bassham Cycle), as displayed in Fig 5 (for more details, see S6 Fig).

The most striking result of this study is that *C*. *necator* produced significant levels of PHB only under IEMC conditions (Fig 4). It is rather contradicting with the fact that *C*. *necator* is known to produce PHB under limited nutrient conditions when using formate as a carbon source [22]. We confirmed the absence of PHB production without electrolysis, regardless of media conditions (I1 and I2), low concentrations of formate (30 mM), relatively high salinity, and nitrogen and TM limitation. The lack of PHB production under non-IEMC conditions in this study is likely because of the relative short time of the experiments, as the literature shows PHB accumulation only at later stages of culturing [23–25]. The relative early production of PHB seen here might be correlated with a faster stress response coupled accumulation of PHB induced by electrolysis.

A possible explanation for the PHB production in IEMC would be the co-generation of hydrogen during the cathodic reaction. Since small quantity of hydrogen was produced in IEMC along with formate, the cytoplasmic soluble hydrogenase (SH) may directly couple H_2_ oxidation with the reduction of NAD^+^ to NADH, producing a higher NADH/NAD^+^ ratio. Moreover, it was reported that PHB producing strains have the ability to balance the reducing equivalents generated from carbon metabolism by using PHB as a sink for reducing power (i.e., NADPH) [26, 27]. Additionally, fermentative bacteria (grown on CO_2_ and H_2_) with high reducing power (NADH) favor metabolite production over cell growth [28]. In opposite to these expectations, the control experiments with introducing hydrogen did not show any PHB production. This result also confirms the hypothesis where electricity and hydrogen must be present at the same time because electricity increases the permeability of the cell membrane via electroporation [29], can be excluded since the IEMC uses only −1.2 V vs. RHE, which is about 1000 times less compared to standard electroporation.

Our remaining hypothesis is the formation of reactive oxygen species (ROS) during the IEMC setup, as its formation was proposed in a similar setup [17]. The ROS toxicity occurred at a similar cathode (In foil) and a potential of –1.6V vs. Ag/AgCl reference electrode, close to the used applied potential in this study. The ROS cause oxidative damage to microorganisms by targeting polypeptide chains in a non-site-specific manner [30]. This might be the reason for the reduction of microbial cell population even when TM and nitrogen were added in media I2, compared to I2 in non-IEMC.

The experimentally observed decline in the microbial cell count during electrolysis, the accumulation of PHB afterwards, the complete lack of PHB production without electrolysis in combination with the knowledge from the literature strongly suggest the presence of reactive oxygen species (ROS) during electrolysis which triggered a cellular stress response that involved the production of PHB. It has been suggested that PHB granules act as a specific site for binding of proteins, which enhances bacterial stress tolerance and resistance to oxidative stress [6, 31–33]. Furthermore, PHB monomers (3HBs) act as chemical chaperones that are capable of protecting enzymes (lipase and lysozyme) from the adverse effects of high oxidation stress [33]. Besides, the solemn occurrence of PHB when electricity was applied to the cells, the harsher media (I1, without TM or nitrogen) promoted higher PHB production than the less harsh media (I2, with TM and nitrogen). This can be explained by the repression of the TCA cycle metabolism, during nitrogen deprivation, resulting in an increase of acetyl-CoA, and this surplus of acetyl-CoA was then redirected from biomass production to the PHB biosynthesis, which is independent of nitrogen [11].

## Conclusions

The one-pot IEMC setup, a combination of In cathode and *C*. *necator*, was attempted to respectively convert CO_2_ to formate, followed by its transformation to PHB. The microbial PHB production was observed only when electrolysis was conducted in the system (at an In cathode potential of −1.2 V vs. RHE). The cogeneration of H_2_ was not the cause that can trigger the PHB production, confirmed by the experiment co-feeding H_2_ as a reactant. The obtained results suggest that ROS induced stress might stimulate PHB production, which is known to have a stress protection function for bioplastic converting bacteria. Increasing the stress conditions for the cells by nitrogen deprivation and a lack of trace metals might further enhance the microbial PHB production.

## Supporting information

S1 Text CO_2_ reduction to formic acid and IEMC. (PDF)

S1 Fig Illustration of a one-pot gas-tight IEMC setup used in this study. (TIF)

S2 Fig Increased current density of the In-NP (indium-nanoparticles deposited) electrode compared to the bare In (indium) electrode, as a function of time (0.1 M KHCO_3_ (pH 6.8) at −1.2 V vs. RHE under CO_2_ bubbling, with Ag/AgCl reference electrode and Pt counter electrode isolated with glass frit). (TIF)

S3 Fig SEM images of the pristine in plate and In-NP deposited electrode. (TIF)

S4 Fig Current density and farradaic efficiency of the In-np electrode. (Left) Current density profiles at different applied potentials as a function of time and (Right) corresponding Faradaic efficiency obtained using In-np electrode (1 × 1.7 cm^2^) in 30 mM potassium phosphate, 100 mM K_2_SO_4_, pH adjusted to 7.5 under CO_2_ bubbling, with Ag/AgCl reference electrode and Pt counter electrode isolated with glass frit.
(TIF)

S5 Fig Electrolysis results for media with different concentrations of either media C or M (see original manuscript for description). The concentration of media C or M was as following: 0, 3 and 50% (v/v), which corresponds, to non, low and high concentrations. Electrochemical reaction performed was (working electrode: In-NP, reference electrode: Ag/AgCl, counter electrode: Pt wire (separated), −1.2 V vs. RHE, CO_2_ saturated, 25°C. 0% of media C (at pH 7.5) or M (at pH 6.8) corresponds to media I1, and 3% of media C (at pH 7.5) or M (at pH 6.8) corresponds to media I2. **A)** Faradaic efficiency at pH 7.5. **B)** Current density at pH 7.5. **C)** Faradaic efficiency at pH 6.8. **D)** Current density at pH 6.8.
(TIF)

S6 Fig The metabolic pathways involved in formate uptake for *M*. *extorquens* AM1. First, it either converts into CO_2_, or ligates with Methylene-THF, before entering the Serine cycle. Then it forms acetyl-CoA and enters the Ethylmalonyl-Coa (EMC) cycle. There, it either branches out in the PHB cycle, or continues through the EMC cycle to regenerate glyoxelate [Peyraud, R. *et al*. Genome-scale reconstruction and system level investigation of the metabolic network of Methylobacterium extorquens AM1. *Bmc Syst Biol* **5**, doi:Artn 18910.1186/1752-0509-5-189 (2011)].
(TIF)
