# Supplementary material for: Poly(3-hydroxybutyrate) production in an integrated electromicrobial setup: Investigation under stress-inducing conditions
Source: PLoS One. 2018 Apr 26;13(4):e0196079. doi: 10.1371/journal.pone.0196079 (PMC5919402; doi:10.1371/journal.pone.0196079)
Supplement: S1 Text — (PDF) [file pone.0196079.s001.pdf]

**S1 Text. $CO_2$ reduction to formic acid and IEMC**

We selected indium (In) as an active electrode selective for formic acid formation, according to the literature [Hori, Y. in *Modern aspects of electrochemistry*, P.89-189 (Springer, 2008)]. An In electrode (1 × 1.7 $cm^2$) as washed with milli-Q and acetone was first tested in 0.1 M $KHCO_3$ (pH 6.8) at −1.2 V vs. RHE and the chronoamperometry result is shown in (S2 Fig). The Faradaic efficiency for formate was as low as 68% under the condition investigated. To improve the Faradaic efficiency, we attempted to deposit indium nanoparticles on the In electrode (denoted as In-np) in two electrode system configuration (counter electrode was also the In electrode). The nanoparticle deposition was conducted under 0.05 M $In_2(SO_4)_3$ and 0.04 M citric acid at −9.3 mA $cm^{-2}$ for 10 min under Ar. S3 Fig shows the surface morphology observed by scanning electron microscopy (SEM), exhibiting that the nanoparticles with approximate sizes of 5-50 nm are uniformly deposited on the In substrate. As can be seen in S2 and S3 Figs, the In-np electrode enhanced the active surface area, resulting in improved current density (per geometric surface area). More interestingly, the Faradaic efficiency for formic acid was improved to 89%, probably because of decoration of unselective sites on the pristine In electrode.

Using the developed In-np electrode, we investigated the dependence of applied potential on current density and Faradaic efficiency. The electrolyte used (I medium; 40 mM potassium phosphate, 100 mM $K_2SO_4$, pH adjusted to 7.5) was selected based on the study optimized for *R. eutropha* (refer to the main manuscript for details). The results are shown in S4 Fig (iR is not corrected, which was measured to be ~10 Ω by impedance measurement). The current density monotonically increased with increasing applied overpotential (S4 Fig left), and intriguingly, the Faradaic efficiency for formic acid also increased with increasing applied overpotential (S4 Fig right). Because larger applied potential (or total voltage) leads to loss of energy efficiency, the optimal applied potential

must be chosen. In this study, we selected −1.2 V vs. RHE to use for further study in IEMC system because reasonable current density of −10 mA $cm^{-2}$ was achieved with relatively high Faradaic efficiency (86%).
