# Supplementary figures and images for: Poly(3-hydroxybutyrate) production in an integrated electromicrobial setup: Investigation under stress-inducing conditions
Source: PLoS One. 2018 Apr 26;13(4):e0196079. doi: 10.1371/journal.pone.0196079 (PMC5919402; doi:10.1371/journal.pone.0196079)

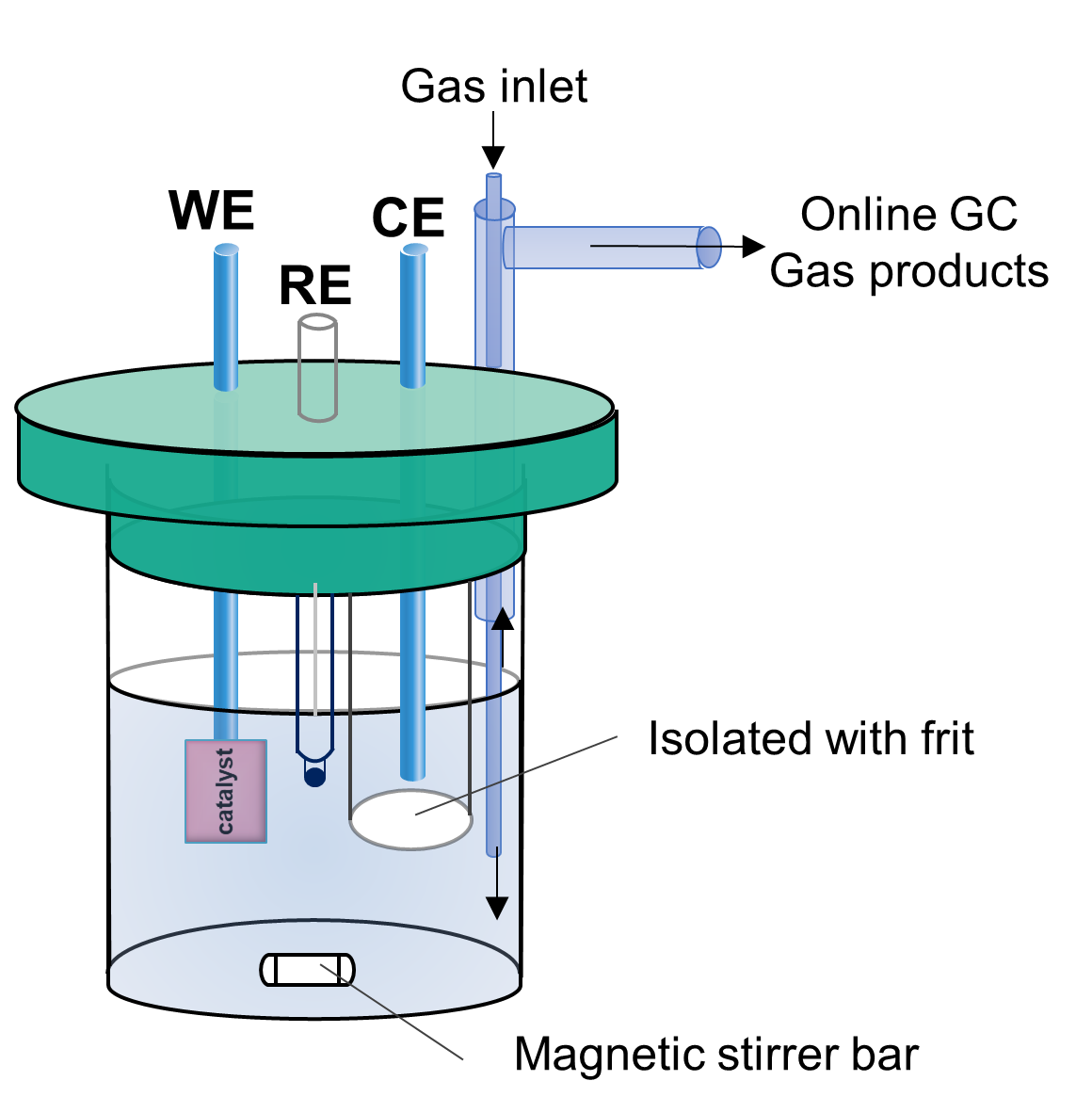

Supplement: S1 Fig — (TIF) [file pone.0196079.s002.tif]

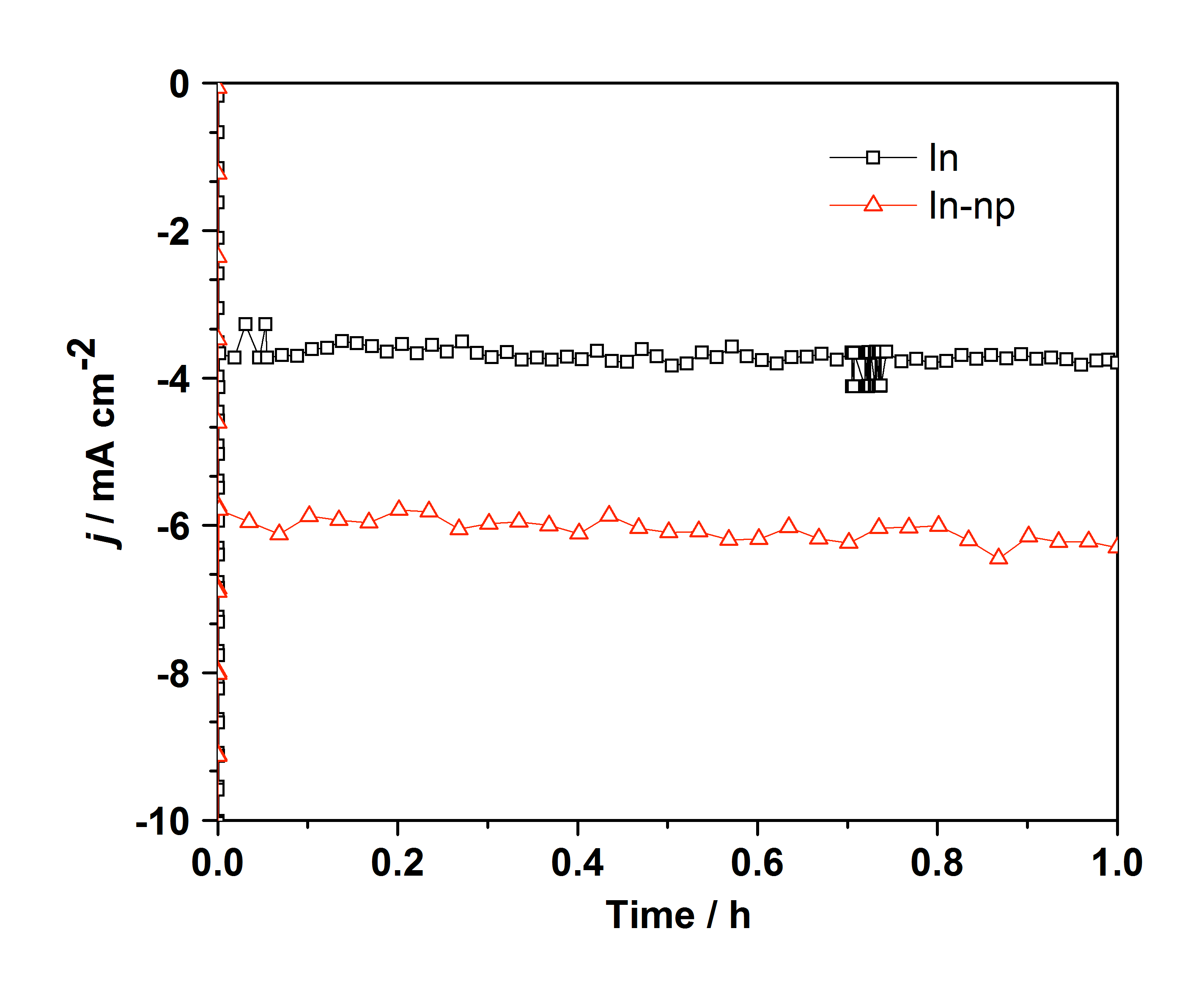

Supplement: S2 Fig — (TIF) [file pone.0196079.s003.tif]

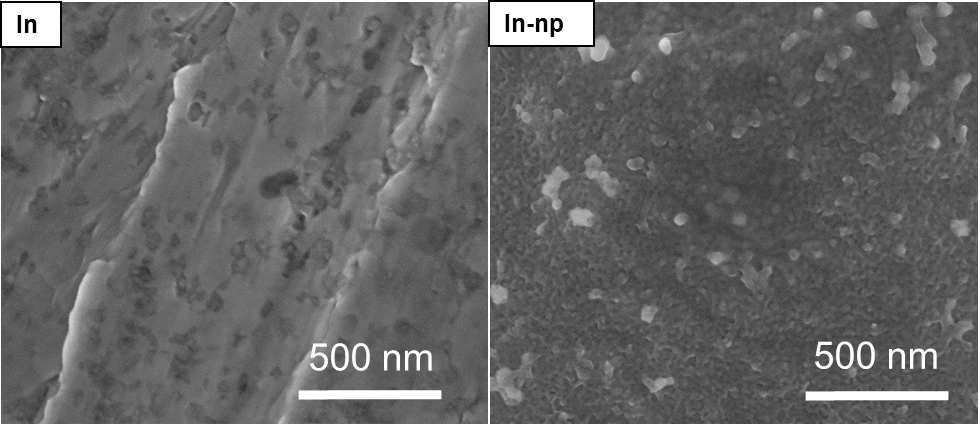

Supplement: S3 Fig — (TIF) [file pone.0196079.s004.tif]

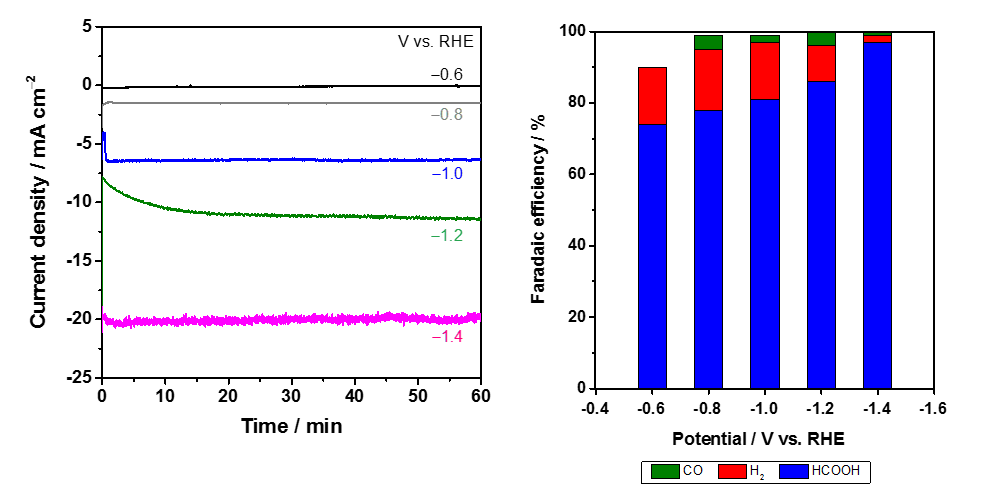

Supplement: S4 Fig — (Left) Current density profiles at different applied potentials as a function of time and (Right) corresponding Faradaic efficiency obtained using In-np electrode (1 × 1.7 cm2) in 30 mM potassium phosphate, 100 mM K2SO4, pH adjusted to 7.5 under CO2 bubbling, with Ag/AgCl reference electrode and Pt counter electrode isolated with glass frit. (TIF) [file pone.0196079.s005.tif]

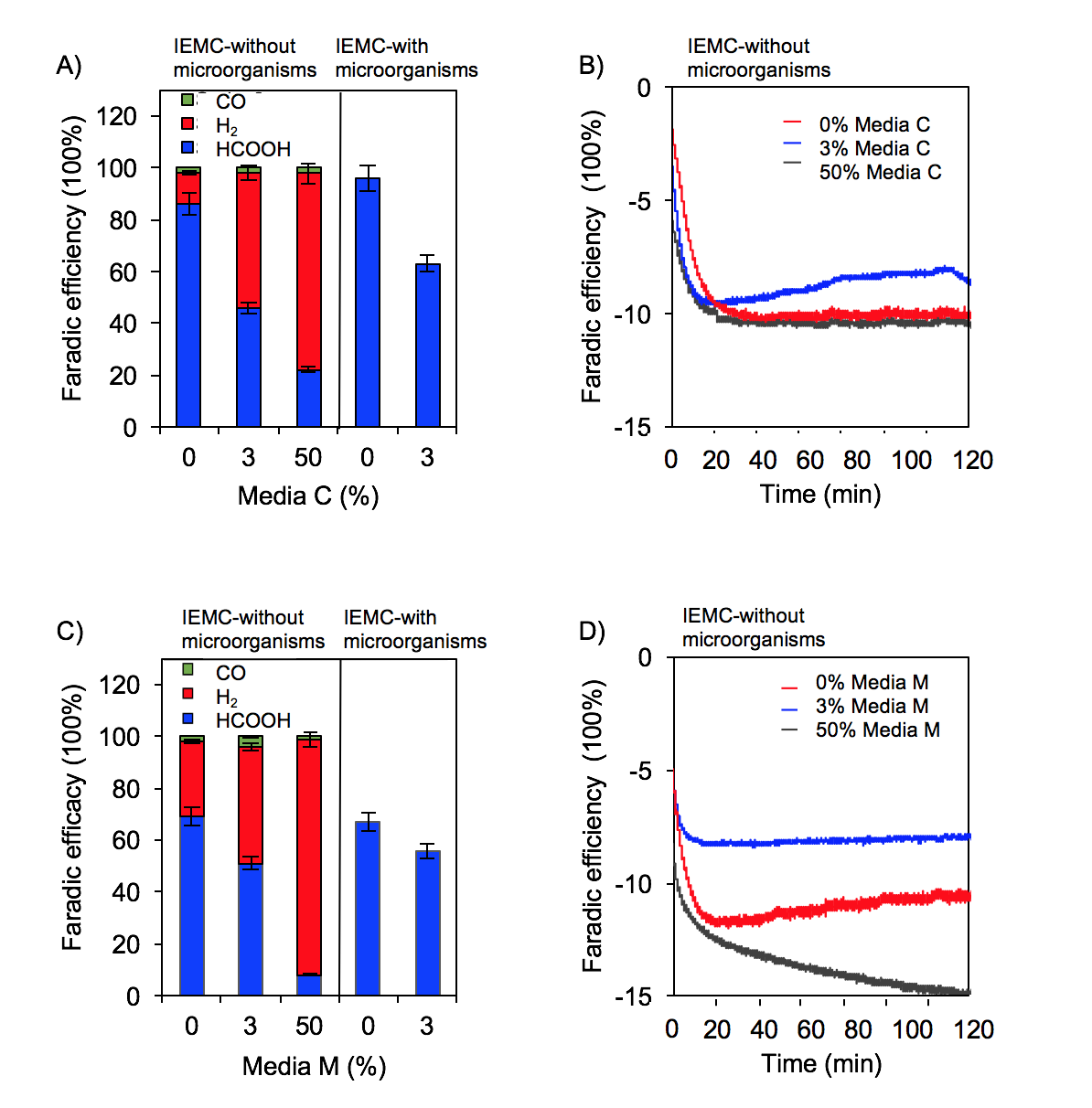

Supplement: S5 Fig — The concentration of media C or M was as following: 0, 3 and 50% (v/v), which corresponds, to non, low and high concentrations. Electrochemical reaction performed was (working electrode: In-NP, reference electrode: Ag/AgCl, counter electrode: Pt wire (separated), −1.2 V vs. RHE, CO2 saturated, 25°C. 0% of media C (at pH 7.5) or M (at pH 6.8) corresponds to media I1, and 3% of media C (at pH 7.5) or M (at pH 6.8) corresponds to media I2. A) Faradaic efficiency at pH 7.5. B) Current density at pH 7.5. C) Faradaic efficiency at pH 6.8. D) Current density at pH 6.8. (TIF) [file pone.0196079.s006.tif]

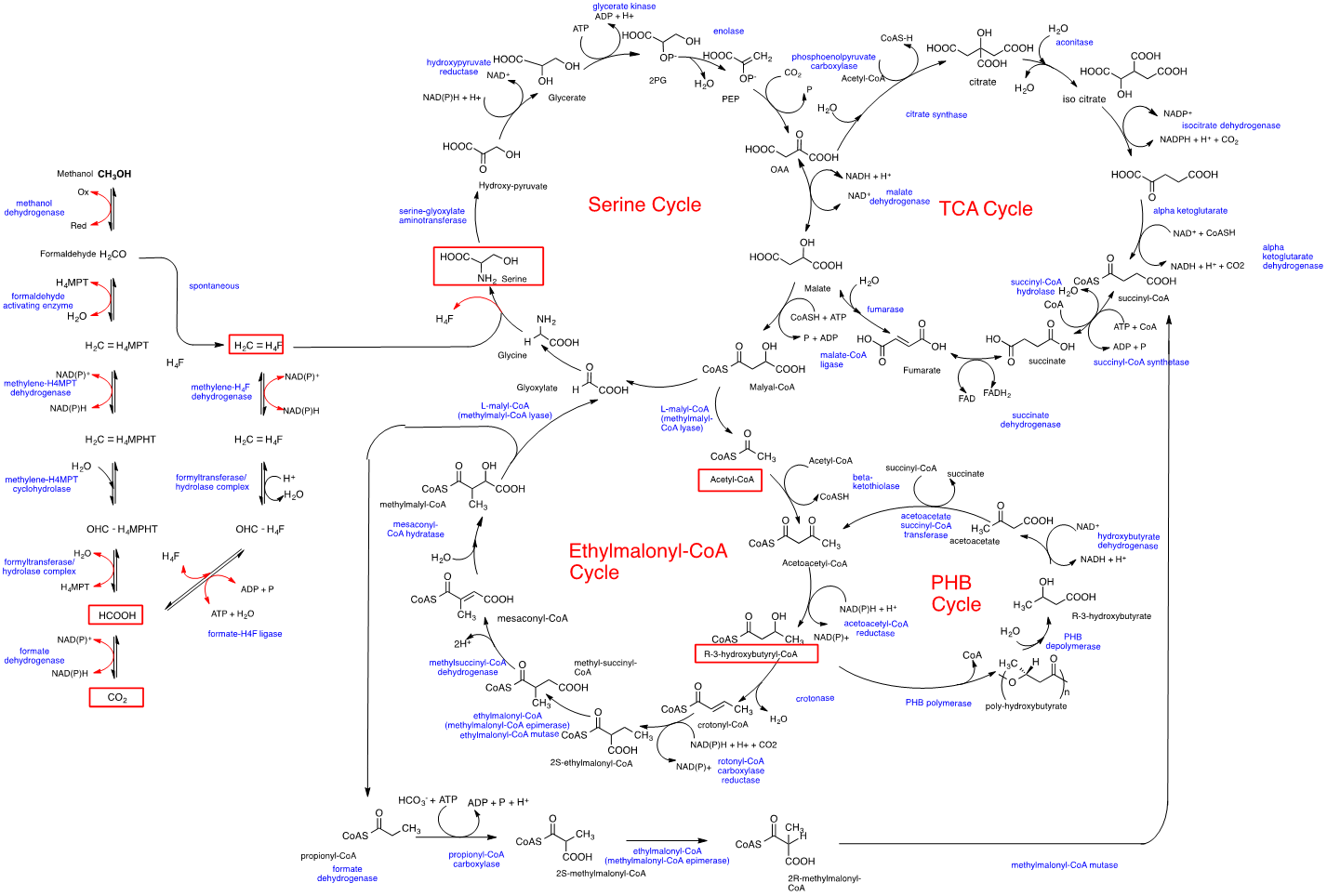

Supplement: S6 Fig — First, it either converts into CO2, or ligates with Methylene-THF, before entering the Serine cycle. Then it forms acetyl-CoA and enters the Ethylmalonyl-Coa (EMC) cycle. There, it either branches out in the PHB cycle, or continues through the EMC cycle to regenerate glyoxelate [Peyraud, R. et al. Genome-scale reconstruction and system level investigation of the metabolic network of Methylobacterium extorquens AM1. Bmc Syst Biol 5, doi:Artn 18910.1186/1752-0509-5-189 (2011)]. (TIF) [file pone.0196079.s007.tif]
